# Association of dietary sodium intake with impaired fasting glucose in adult cancer survivors: A population-based cross-sectional study

**DOI:** 10.1371/journal.pone.0286346

**Published:** 2023-05-25

**Authors:** Kyuwoong Kim, Hamee Kim, Tae Joon Jun, Young-Hak Kim

**Affiliations:** 1 National Cancer Control Institute, National Cancer Center, Goyang, Republic of Korea; 2 Department of Medical Informatics, Kyung Hee University Medical Center, Seoul, Republic of Korea; 3 Big Data Research Center, Asan Institute for Life Science, Asan Medical Center, Seoul, Republic of Korea; 4 Division of Cardiology, Department of Internal Medicine, Asan Medical Center, University of Ulsan College of Medicine, Seoul, Republic of Korea; Kyung Hee University School of Medicine, REPUBLIC OF KOREA

## Abstract

**Background:**

Dietary sodium intake is a crucial lifestyle factor that should be assessed in adult cancer survivors due to their increased risk of adverse health outcomes compared to the general population. However, its with impaired fasting glucose (IFG) in adult cancer survivors remains unclear. This study aimed to investigate the association of dietary sodium intake categorized by the American Heart Association (AHA) recommendation with IFG in the community-dwelling adult cancer survivors.

**Methods:**

A total of 1,052 adult cancer survivors without diabetes were identified from the sixth and seventh Korea National Health and Nutrition Examination Survey (KNHANES), 2013–2018. Data on dietary sodium intake was categorized as <1,500 mg/day, 1,500–2,999 mg/day, 2,300–3,999 mg/day, and ≥4,000 mg/day according to the AHA recommendation. A multiple logistic regression model adjusted for demographic, lifestyle, and health status was used to compute odds ratios (OR) and 95% confidence intervals (95% CI) for IFG according to dietary sodium intake categories.

**Results:**

After adjusting for confounding variables identified in the KNHANES, the adjusted OR among the adult cancer survivors who consumed 1,500–2,999 mg/day, 2,300–3,999 mg/day, and ≥4,000 mg/day of dietary sodium were 1.16 (95% CI: 0.25–5.27), 1.93 (95% CI: 0.40–9.37), and 2.67 (95% CI: 0.59–12.18), respectively, as compared to those who consumed <1,500 mg/day (*P* value for trend = 0.036).

**Conclusion:**

Among community-dwelling adult cancer survivors, high dietary sodium intake was marginally associated with increased odds of IFG. Well-designed cohort studies or randomized clinical trials are needed to establish more epidemiologic evidence on this association in adult cancer survivors.

## Introduction

The American Heart Association (AHA) recommends that adults should not take more than 2,300 milligrams (mg) of sodium per day and ideally limit the intake of dietary sodium to less than 1,500 mg per day for substantial health benefits. In addition, the *American Cancer Society (ACS)* stated the importance of nutrition after cancer diagnosis and treatment to avoid adverse health outcomes such as cancer recurrence, cardiovascular disease (CVD), and mortality in the *ACS Nutrition and Physical Activity Guideline for Cancer Survivors [1]*. However, limited information is available on the association between dietary sodium intake and impaired fasting glucose (IFG), a common metabolic disorder that could potentially progress to diabetes, leading to adverse health outcomes in cancer survivors [2–11].

Most studies examining dietary sodium intake and glycemic disorder have focused on the association between sodium status and new-onset type 2 diabetes (T2DM). A recent meta-analysis of 44 observational studies including more than 500,000 participants reported that sodium intake was significantly higher (weight mean difference = 621.7 mg/day; 95% confidence intervals [CI]: 321.5 to 922.1) in individuals with T2DM than the non-diabetic controls suggesting the importance of limiting dietary sodium intake for prevention of T2DM [12]. In addition, well-established studies have shown the association of IFG and T2DM with 1- to 4- fold increased risk of CVD, all-cause, and cause-specific mortality in the general population and adult cancer survivors [13–18]. Despite the clinical importance of glycemic control in adult cancer survivors, a paucity of data is available on the association between dietary sodium intake and IFG, among cancer survivors.

This study aimed to investigate the association of dietary sodium intake categorized as the AHA recommendation with IFG among community-dwelling adult cancer survivors. We hypothesized that higher dietary sodium intake would be positively associated with higher odds of IFG among adult cancer survivors.

## Materials and methods

### Study population, design, and data collection

**Fig 1:**
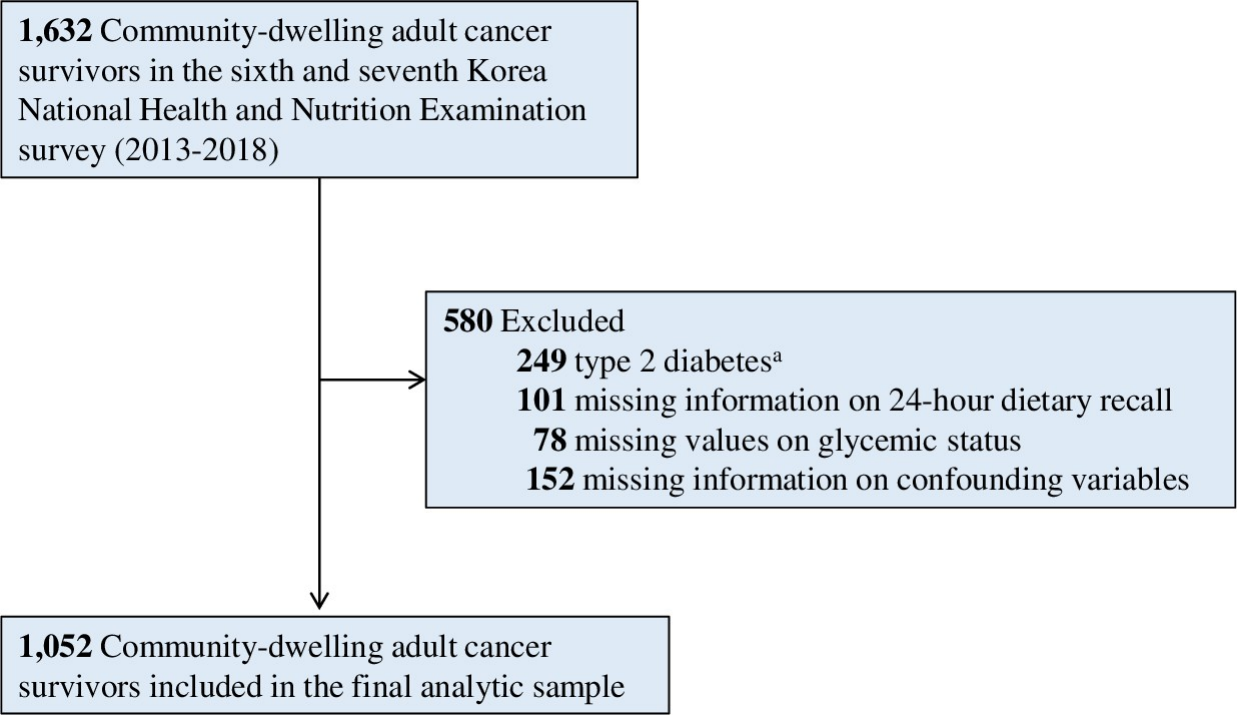
Flowchart of the study population. ^a^Fasting serum glucose ≥126 mg/dL or self-reported diagnosis of diabetes.

Among the participants of the sixth and seventh Korea National Health and Nutrition Examination Survey (KNHANES 2013–2018), we abstracted data on community-dwelling adult cancer survivors based on the self-reported questionnaire on the history of cancer diagnosis. The KNHANES was established by the Korea Disease Control and Prevention Agency (KDCA) in 1998 to assess the health and nutrition status of noninstitutionalized civilians in the Republic of Korea. The KNHANES is an ongoing, nationally representative surveillance project with a complex, multi-stage probability sample design that has been used for a wide range of epidemiologic studies including adult cancer survivors [19–23]. Details of the KNHANES have been described in previous studies [24–26]. After identifying adult cancer survivors in the KNHANES 2013–2018 dataset (n = 1,632), we excluded those with type 2 diabetes (n = 249), missing information on dietary assessment (n = 101), glycemic status (n = 78), and confounding variable (n = 152) to examine the association of dietary sodium intake with IFG in adult cancer survivors (Fig 1). Participants of the sixth and seventh KNHANES (2013–2018) provided informed consent prior to enrolment into the study. The Institutional Review Board of the KDCA approved KNHANES (2013–2018) for providing the dataset in anonymized form for public health research (2013-12EXP-03-5C). Only authorized individuals who were granted permission by the KDCA had access to the dataset. This study was carried out in accordance to the ethical principles of the Declaration of Helsinki. Detailed information on the KNHANES can be found on the official website of the KDCA (www.kdca.go.kr).

### Assessment of dietary sodium intake

Of the adult cancer survivors included in the final study population, dietary sodium intake (mg/day) was assessed with a self-administered 24-hour recall questionnaire as a part of the KNHANES survey series on nutrition status. According to the AHA recommendation on the ideal limit, dietary sodium intake among adult cancer survivors was categorized into <1,500 mg/day, 1,500~2,299 mg/day, 2,300~3,999 mg/day, and ≥4,000 mg/day. Details on the measurement method and validity of the self-administered 24-hour dietary recalls of the KNHANES are described in detail elsewhere. Additionally, a previous study showed high correlation between dietary assessment with the 24-hour recall method and Food Frequency Questionnaire (FFQ) for usual portion size and amount of daily consumption among various food groups in the KNHANES [27].

### Ascertainment of impaired fasting glucose

Blood samples were collected from the adult cancer survivors who participated in the KNHANES and laboratory tests were conducted to provide further information on their health status. IFG was defined as fasting plasma glucose (FPG) of 100–125 mg/dL (5.6–6.9 mmol/L) or glycated hemoglobin (HbA1c) levels of 5.7%–6.4% according to the Clinical Practice Guidelines for Diabetes Mellitus of the Korean Diabetes Association (KDA) [28]. The KDA criteria for IFG are in line with the American Diabetes Association (ADA), which recommends using both FPS and HbA1c for the diagnosis of IFG.

### Assessment of confounding variables

Information on sociodemographic factors and health behavior was obtained from the self-administered survey in the KNHANES. The following variables were collected: age (categorized as <65 years and ≥65 years), sex (male and female), education level (elementary school, middle school, high school, and college/university), household income (defined as the total sum of income divided by the square root of the number of family members for each household and categorized into quartiles), cigarette smoking (current smoker, past smoker, and never smoker), alcohol consumption (non-drinker and drinker), and physical activity (aerobic exercise, muscle-strengthening exercise, and balance and flexibility exercise).

Measurement on the noninvasive anthropometric measure (waist circumference [WC]) and blood pressure (brachial systolic blood pressure [SBP] and diastolic blood pressure [DBP]) was conducted by professionally trained medical staff and lipid profile (high-density lipoprotein cholesterol [HDL-C] and triglyceride [TG]) was measured from laboratory analysis of blood samples. Total energy intake and macro-and-micro nutrient intake (carbohydrate, fat intake, protein intake, potassium intake) were obtained from self-administered 24-hour dietary recall survey. Multi-collinearity between the confounding factors was visually assessed.

### Statistical analysis

Sociodemographic, health behavior, health and nutrition status among adult cancer survivors across the categories of dietary sodium intake defined by the AHA recommendation were compared with survey-weighted regression and Rao-Scott F-adjusted chi-square test for continuous and categorical variables, respectively [29, 30]. We computed means (standard error, SE) and numbers (percentages) in each continuous and categorical variable using the sample weights reflecting complex, multi-stage probability sample design of the KNHANES. To investigate the association of dietary sodium intake based on the AHA recommendation categories and IFG, we used multiple logistic regression to compute adjusted odds ratios (OR) and 95% CI [31]. First, we constructed a minimally adjusted model (adjusted for age, sex, and physical activity) as Model 1 and further adjusted for sociodemographic factors (household income and education level) and health behavior (cigarette smoking and alcohol consumption) (Model 2). The fully adjusted model (Model 3) was adjusted for dietary factors (total energy intake, fat intake, protein intake, carbohydrate intake, and potassium intake) in addition to the variables included in Model 1 and Model 2. The linear trend for odds of IFG across the categories of dietary sodium intake was also tested. To visually examine the relationship between dietary sodium intake and IFG, we created restricted cubic spline plots using the fully adjusted multiple logistic regression model (Model 3). Data collection and analyses in this study were conducted using SAS software version 9.4 (SAS Institute., NC, USA). Visualization of multicollinearity was performed with Python 3.6. We set the statistical significance at *P* values less than 0.05.

## Results

### Characteristics of the study population

Among 1,052 adult cancer survivors, only 16.6% were taking less than 1,500 mg of sodium per day, which was suggested as the ideal limit of daily sodium intake by the AHA for individuals at risk of hypertension or cardiovascular disease. The median (interquartile range [IQR]) for dietary sodium intake across the dietary sodium intake categories were 1,093 mg/day (IQR: 793–1,323), 1,878 mg/day (IQR: 1,694–2,101), 2,995 mg/day (IQR: 2,644–3,449), 5,192 mg/day (IQR: 4,409–6,461), respectively. Adult cancer survivors in the highest category of dietary sodium intake (≥4,000 mg/day) were relatively younger (less than 65 years of age), and had a higher level of nutrition intake. Overall, health behavior and health status among adult cancer survivors did not significantly differ across the increasing levels of dietary sodium intake categories except for cigarette smoking and WC (Table 1).

**Table 1:** Characteristics of adult cancer survivors according to dietary sodium intake in the Korea National Health and Nutrition Examination Survey VI and VII (2013–2018).

	Dietary sodium intake^a^				
Characteristics	<1,500 mg/day (*N* = 175)	1,500–2,299 mg/day (*N* = 232)	2,300–3,999 mg/day (*N* = 389)	≥4,000 mg/day (*N* = 256)	*p*-value^b^
Dietary sodium intake g/day (median, IQR)	1,093 (793–1,323)	1,878 (1,694–2,101)	2,995 (2,644–3.449)	5,192 (4,409–6,461)	
Age					
<65 years	79 (45.1)	129 (55.6)	218 (56.0)	159 (62.1)	0.022
≥65 years	96 (54.9)	103 (44.4)	171 (44.0)	97 (37.9)	
Sex					
Male	36 (20.6)	63 (27.2)	148 (38.1)	112 (43.8)	<0.001
Female	139 (79.4)	169 (72.8)	241 (62)	144 (56.3)	
Household income^c^					
1Q	65 (36.6)	63 (27.2)	92 (23.7)	54 (21.1)	0.005
2Q	42 (24.0)	74 (31.9)	96 (24.7)	69 (27.0)	
3Q	41 (23.4)	47 (20.3)	98 (25.3)	66 (25.8)	
4Q	28 (16.0)	48 (20.7)	102 (26.3)	67 (26.2)	
Education level					
Elementary school	76 (43.9)	83 (36.1)	118 (30.4)	71 (27.8)	<0.001
Middle school	26 (14.5)	36 (15.2)	51 (13.1)	32 (12.6)	
High school	42 (24.3)	61 (26.5)	120 (30.9)	75 (29.4)	
College/University	30 (17.3)	51 (22.2)	100 (25.5)	80 (30.2)	
Cigarette smoking					
Current smoker	6 (3.4)	19 (8.2)	31 (8.0)	17 (6.6)	<0.001
Past smoker	33 (18.9)	47 (20.3)	111 (28.5)	86 (33.6)	
Never smoker	136 (77.7)	166 (71.6)	247 (63.5)	153 (59.8)	
Alcohol consumption					
Non-drinker	46 (26.3)	63 (27.2)	102 (26.2)	69 (27.0)	0.929
Drinker	129 (73.7)	169 (72.8)	287 (73.8)	187 (73.1)	
Physical activity					
Aerobic exercise	57 (35.6)	85 (43.6)	149 (42.5)	106 (48.6)	0.051
Muscle-strengthening exercise	31 (17.7)	50 (21.6)	99 (25.5)	73 (28.5)	0.274
Balance and flexibility exercise	30 (17.1)	60 (25.9)	85 (21.9)	77 (30.1)	0.036
Metabolic health (SE)					
WC, cm	80.5 (0.6)	79.9 (0.8)	81.3 (0.6)	82 (0.6)	0.038
SBP, mmHg	122.2 (1.6)	121.8 (1.3)	119.3 (0.9)	122.7 (1.2)	0.657
DBP, mmHg	75.7 (0.8)	75.6 (0.7)	74.6 (0.6)	76.2 (0.7)	0.612
HDL-C, mg/dL	52 (0.9)	52 (1.0)	51.7 (0.8)	50.4 (0.8)	0.232
TG, mg/dL	111 (4.0)	129.7 (7.3)	117.6 (3.7)	129.6 (7.0)	0.299
FPG, mg/dL	95.3 (0.7)	96.3 (0.7)	96.3 (0.7)	97 (0.7)	0.565
Total energy intake, mean (SE), kcal/day	1221.7 (56.5)	1480.5 (34.5)	1873.1 (32)	2375.5 (55.3)	<0.001
Carbohydrate intake, g/day	219.6 (11.7)	255 (6.8)	306.2 (5.7)	373.3 (7.5)	<0.001
Fat intake, g/day	20.2 (1.4)	26.2 (1.2)	37.8 (1.3)	47.5 (2.3)	<0.001
Protein intake, g/day	37.9 (1.8)	48 (1.3)	66.2 (1.6)	90.1 (3.3)	<0.001
Potassium intake, mg/day	2213.8 (228.0)	2461.3 (89.4)	3117 (81.3)	4017.9 (139.1)	<0.001

NOTE: Values above are presented as n (%) unless otherwise specified

^a^Categorized according to the AHA recommendation

^b^Calculated from Rao-Scott Chi-Square Test for categorical variables and survey-weighted regression for continuous variables, respectively

^x^Proxy for socioeconomic status (calculated from the sum of the income from the members of each household divided by the square root of the number of household members)

Acronyms/Abbreviations: IQR, interquartile range; SE, standard error; WC, waist circumference; SBP, systolic blood pressure; DBP, diastolic blood pressure; HDL-C, high density lipoprotein cholesterol; TG, triglyceride; FPG, fasting plasma glucose

### Association between dietary sodium intake and impaired fasting glucose in adult cancer survivors

**Fig 2:**
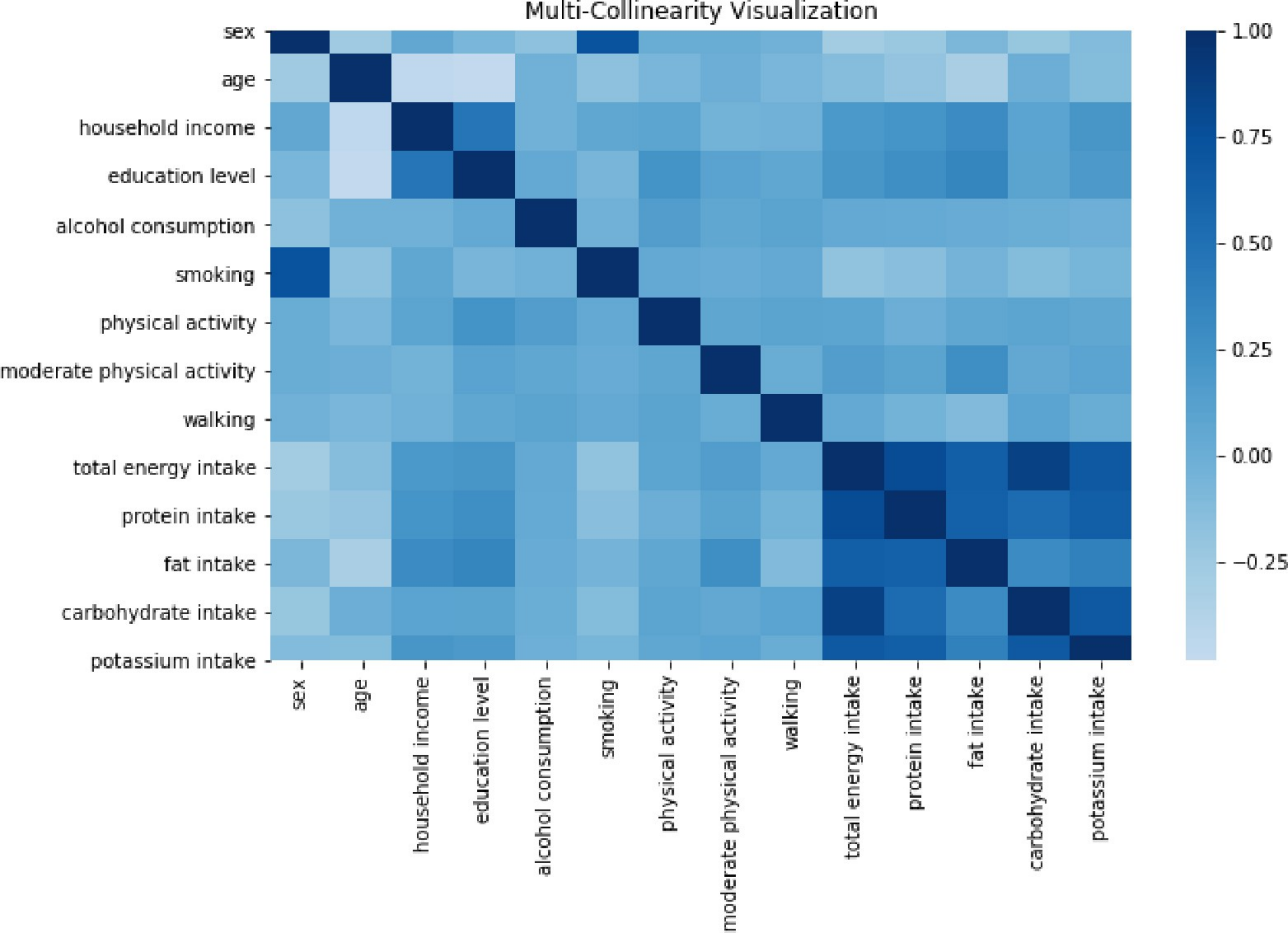
Representation of multicollinearity between the variables used to assess dietary sodium intake and impaired fasting glucose in adult cancer survivors.

**Fig 3:**
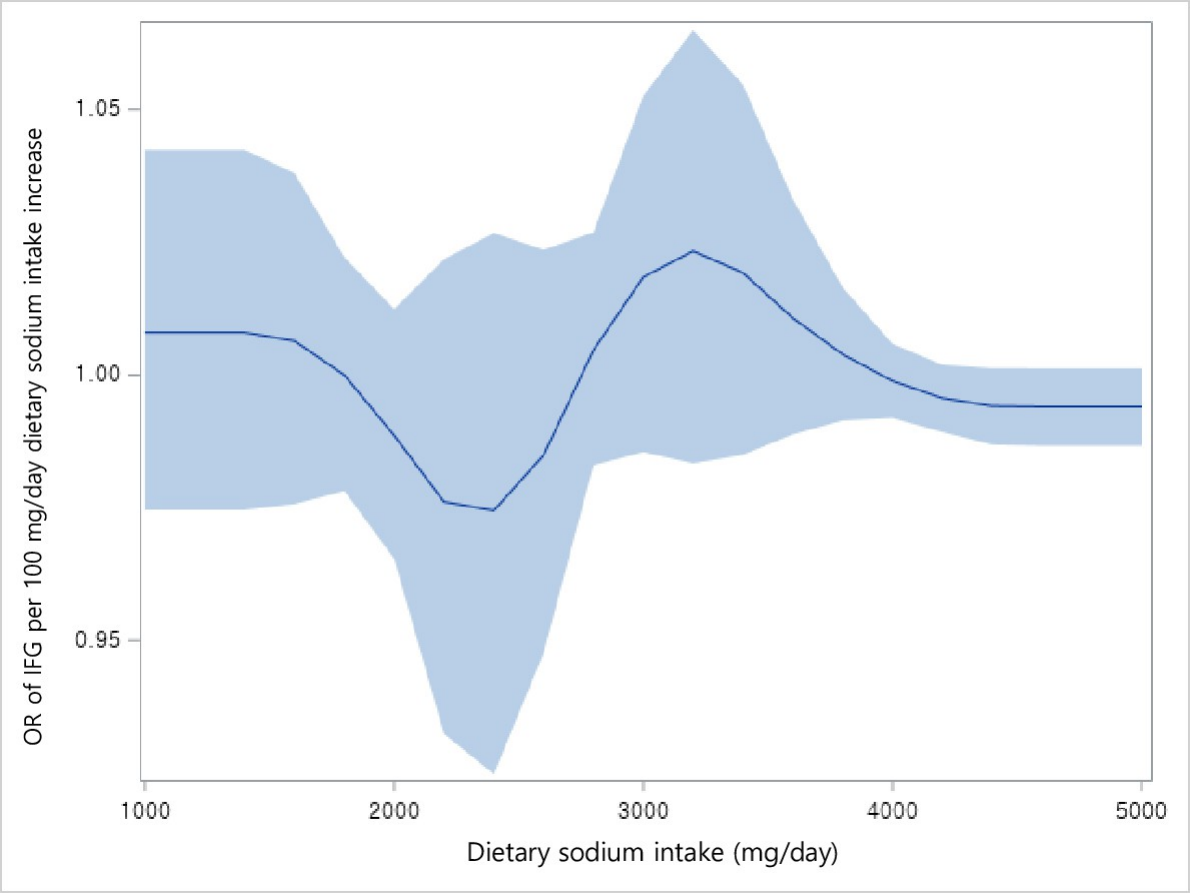
Restricted cubic splines displaying adjusted odds ratios of impaired fasting glucose with 95% confidence intervals according to 100 mg/day dietary sodium intake increase. Odds ratios (OR) were calculated by multivariable logistic regression adjusted for age, sex, physical activity, household income, education level, cigarette smoking, alcohol consumption, total energy intake, fat intake, protein intake, carbohydrate intake, and potassium intake.

Of the 1,052 adult cancer survivors, 583 had IFG (55.4%). Compared to the lowest category of dietary sodium intake (<1,500 mg/day), the odds of IFG across 1,500–2,299 mg/day, 2,300–3,999 mg/day, and ≥4,000 mg/day of dietary sodium intake were 1.16 (95% CI: 0.25–5.27), 1.93 (95% CI: 0.40–9.37), and 2.67 (95% CI: 0.59–12.18), respectively, after adjusting for age, sex, physical activity, household income, education level, cigarette smoking, alcohol consumption, total energy intake, fat intake, protein intake, carbohydrate intake, and potassium intake (Model 3). Similar associations were found in the minimally adjusted model (Model 1 adjusted for age, sex, and physical activity) and Model 2 (adjusted for household income, education level, cigarette smoking, and alcohol consumption in addition to Model 1) (Table 2). Among these variables used for adjusted analyses, multi-collinearity was relatively higher between micro-and-macro nutrient intake (i.e., fat intake, protein intake, carbohydrate intake, and potassium intake) compared to other variables (Fig 2). Linear trends for the odds of IFG across the categories of dietary sodium intake were statistically significant (*P* for trend = 0.004 in Model 1, *P* for trend = 0.014 in Model 2, *P* for trend = 0.036 in Model 3, respectively). Restricted cubic spline plots showed that the odds of IFG per 100 grams per day in dietary sodium intake showed marginally increasing trends around 2,500 mg per day up to 3,000 mg per day (Fig 3).

**Table 2:** Association between dietary sodium intake and prediabetes among adult cancer survivors in the Korea National Health and Nutrition Examination Survey VI and VII (2013–2018).

	Dietary sodium intake^a^				
Variable	<1,500 mg/day (*N* = 175)	1,500–2,299 mg/day (*N* = 232)	2,300–3,999 mg/day (*N* = 389)	≥4,000 mg/day (*N* = 256)	*p*-value for trend
Dietary sodium intake, mg/day (median, IQR)	1,090 (828–1,304)	1,871 (1,693–2,101)	3,020 (2,658–3.462)	5,190 (4,407–6,464)	
No. of cases	100	117	224	142	
Model 1: minimally adjusted OR (95% CI)	1 (Reference)	1.49 (0.44–6.70)	2.29 (0.50–10.51)	3.03 (0.67–13.82)	0.004
Model 2: multivariable-adjusted OR (95% CI)	1 (Reference)	1.42 (0.28–7.31)	2.28 (0.44–11.76)	2.73 (0.57–13.07)	0.014
Model 3: multivariable-adjusted OR (95% CI)	1 (Reference)	1.16 (0.25–5.27)	1.93 (0.40–9.37)	2.67 (0.59–12.18)	0.036

NOTE: OR (95% CI) presented above are weighted according to the national adult population of the KNHANES (2013–2018)

^a^Categorized according to the AHA recommendation

Model 1: Multiple logistic regression model adjusted for age, sex, and physical activity

Model 2: Multiple logistic regression model adjusted for age, sex, physical activity and additional sociodemographic factors (household income and education level) and health behavior (cigarette smoking and alcohol consumption)

Model 3: Multiple logistic regression model adjusted for variables included in Model 2 and dietary factors (total energy intake, fat intake, protein intake, carbohydrate intake, and potassium intake)

Acronyms/Abbreviations: AHA, American Heart Association; IQR, interquartile range; OR, odds ratio; CI, confidence interval; KNHANES, Korea National Health and Nutrition Examination Survey

## Discussion

In this population-based, cross-sectional analysis of community dwelling adult cancer survivors, we found a marginal association between dietary sodium intake categorized by the AHA recommendation and IFG. As compared to the ideal limit of dietary sodium intake recommended by the AHA (i.e., less than 1,500 mg per day), the odds of IFG were approximately 1.2 to 2.7 times higher in high consumption categories. While these estimates from categorical analysis showed statistically nonsignificant results, a linear trend for odds of IFG across the categories of dietary sodium intake was observed.

The findings of this study are similar to the previous observational studies and randomized, controlled trial examining the association between dietary sodium intake and T2DM in the general population and dietary sodium intake in patients with T2DM, respectively. Rush *et al* showed in a cross-sectional analysis that the prevalence of diabetes was almost 2-fold higher in the community-dwelling US adults who belong to the highest quartile of calorie-adjusted daily intake of sodium compared to those in the lowest quartile [32]. Compared to the nutrient residual method used to derive the calorie-adjusted daily sodium intake in that study, our study directly assessed the association between dietary sodium intake categories according to the AHA recommendations and IFG and included total energy intake as a confounding variable in the analysis. In the Enhancing Adherence in Type 2 Diabetes (ENHANCE) trial that included 251 US adults with T2DM to test the efficacy of lifestyle management, only about 20% of the patients adhered to the AHA recommendation for consuming less than 2,300 mg of sodium per day and about 2% of met the criteria for the ideal limit of less than 1,500 mg per day [33].

Similar to the results of the ENHANCE trial, a longitudinal Australian study with 782 patients with T2DM with data on dietary sodium intake estimated from 24-hour urinary excretion of sodium showed that most patients with T2DM do not meet the dietary sodium guideline recommended by the Australian National Heart Foundation (<2,300 mg/day), which is in line with the AHA recommendation (i.e., no more than 2,300 mg/day and ideally limit the consumption to less than 1,500 mg/day) [34]. While these studies demonstrated the importance of lowering dietary sodium for the prevention of T2DM and the status of nonadherence to the dietary guidelines in patients with T2DM, our findings add to the current evidence that limiting dietary sodium intake according to the AHA recommendation might also be of clinical importance for preventing IFG among adult cancer survivors.

Of note, glycemic control of patients with IFG should be addressed as a public health concern. In a recent meta-analysis of 129 prospective cohort studies or post-hoc analysis of clinical trials including more than 10 million individuals, IFG was associated with a 13% increase in all-cause mortality and 15% increase in composite CVD outcomes compared to those with normal glucose level [35]. Also, IFG before cancer diagnosis was marginally associated with secondary primary cancer (HR = 1.21; 95% CI: 0.75–1.96) in a large cohort of male cancer survivors [13]. Taking the previous evidence and findings of this study together, the management of glycemic disorder partially through reducing sodium intake according to the AHA recommendation should be considered for population-level strategy for preventing adverse health outcomes in cancer survivors. As described earlier, our categorical analyses showed statistically nonsignificant results and only the linear trend reached the statistical significance cut-off point. In this regard, some researchers pointed out in a systematic review of well-regarded oncology literature that the *p*-value threshold alone could not entirely conclude the importance of the findings and the findings should not be solely based on this threshold [36]. Nonetheless, well-established observational studies or randomized clinical trials including adult cancer survivors are needed to establish more evidence.

The following limitations should be considered when interpreting the findings of our study. First, our study sample of community-dwelling adult cancer survivors only selected those who participated in the KNHANES (2013–2018) with a relatively small sample size (n = 1,052), which may limit generalizability. Second, our analyses were based on a cross-sectional data examining the correlation between dietary sodium intake and IFG without follow-up period. Thus, the causal inference could not be derived from this cross-sectional analysis. Third, we were only able to assess dietary sodium intake level through self-reported dietary behavior at a single time-point without further validation or assessment of changes in dietary behavior. However, such information is rarely available in a nationally representative cross-sectional study. Lastly, our study subjects included adult cancer survivors within the Korean population. Thus, the findings of this study should be tested in multi-ethnic cohort of adult cancer survivors. Despite these limitations, our study demonstrates the potential role of dietary sodium intake level categorized by the AHA recommendation in association with IFG among adult cancer survivors. Considering these limitations and findings of the current study, future research that includes follow-up information on dietary intake and glycemic levels in an ethnically diverse population of cancer survivors is warranted. Such well-designed prospective studies would be essential to better understand the association of dietary sodium intake with IFG in adult cancer survivors.

## Conclusion

In this population-based, cross-sectional study among community-dwelling adult cancer survivors, dietary sodium intake above the AHA recommendation level (more than 2,300 g/day) was marginally associated with higher odds of IFG. Further studies are needed to examine adherence to evidence-based dietary guidelines and health outcomes among cancer survivors.
