# Correction: Association of dietary sodium intake with impaired fasting glucose in adult cancer survivors: A population-based cross-sectional study

**DOI:** 10.1371/journal.pone.0314645

**Published:** 2024-11-21

**Authors:** Kyuwoong Kim, Haemee Kim, Tae Joon Jun, Young-Hak Kim

The second author’s name is spelled incorrectly. The correct name is: Haemee Kim.

In the Funding statement, the grant number from the funder Ministry of Health and Welfare, Republic of Korea is listed incorrectly. The correct grant number is: HR21C0198.
