# Correction: Correction: Association of dietary sodium intake with impaired fasting glucose in adult cancer survivors: A population-based cross-sectional study

**DOI:** 10.1371/journal.pone.0318187

**Published:** 2025-01-23

**Authors:** The PLOS ONE Staff

## Notice of Republication

This article was republished on January 14, 2025, to correct an error in the third author’s name that was introduced during the typesetting process. The publisher apologizes for the errors. Please download this article again to view the correct version. The originally published, uncorrected article and the republished, corrected articles are provided here for reference.

## Supporting information

S1 File Originally published, uncorrected article. (PDF)

S2 File Republished, corrected article. (PDF)
