# Supplementary material for: Correction: Correction: Association of dietary sodium intake with impaired fasting glucose in adult cancer survivors: A population-based cross-sectional study
Source: PLoS One. 2025 Jan 23;20(1):e0318187. doi: 10.1371/journal.pone.0318187 (PMC11756762; doi:10.1371/journal.pone.0318187)
Supplement: S2 File — (PDF) [file pone.0318187.s002.pdf]

PLOS ONE

CORRECTION

# Correction: Association of dietary sodium intake with impaired fasting glucose in adult cancer survivors: A population-based cross-sectional study

Kyuwoong Kim, Haemee Kim, Tae Joon Jun, Young-Hak Kim

The second author's name is spelled incorrectly. The correct name is: Haemee Kim.

In the Funding statement, the grant number from the funder Ministry of Health and Welfare, Republic of Korea is listed incorrectly. The correct grant number is: HR21C0198.

## Reference

1. Kim K, Kim H, Jun TJ, Kim Y-H (2023) Association of dietary sodium intake with impaired fasting glucose in adult cancer survivors: A population-based cross-sectional study. PLOS ONE 18(5): e0286346. https://doi.org/10.1371/journal.pone.0286346 PMID: 37228155

Check for updates

OPEN ACCESS

**Citation:** Kim K, Kim H, Jun TJ, Kim Y-H (2024) Correction: Association of dietary sodium intake with impaired fasting glucose in adult cancer survivors: A population-based cross-sectional study. PLoS ONE 19(11): e0314645. https://doi.org/10.1371/journal.pone.0314645

**Published:** November 21, 2024

**Copyright:** © 2024 Kim et al. This is an open access article distributed under the terms of the Creative Commons Attribution License, which permits unrestricted use, distribution, and reproduction in any medium, provided the original author and source are credited.
